# Deficiency and Development: A Bibliometric Analysis of the Effects of Iron Deficiency on Human Development

**DOI:** 10.7759/cureus.92544

**Published:** 2025-09-17

**Authors:** Mehreen Mohammed, Meghana Konda, Latha Ganti

**Affiliations:** 1 Nutrition, Granite Bay High School, California, USA; 2 Public Health, Brown University, Providence, USA; 3 Emergency Medicine & Neurology, University of Central Florida, Orlando, USA; 4 Medical Science, The Warren Alpert Medical School of Brown University, Providence, USA

**Keywords:** anemia, bibliometric analysis, global research trends, human development, iron deficiency, nutrition, pediatric health

## Abstract

Iron is an important micronutrient for biological and neurocognitive function. Iron deficiency is significantly undertreated; yet, for many vulnerable populations, such as women and children, iron deficiency is prevalent and leads to serious developmental consequences. As global trends continue to rise, it is crucial to research trends in this area.

Publications were extracted from the Web of Science (WoS) Core Collection database, specifically containing the keywords “iron deficiency” and “development.” A total of 6,850 articles were acquired from the search. A bibliometric analysis was then conducted on VOSViewer 1.6.2 to assess data concerning the publication year, country, journal, and keyword frequency.

The quantity of published articles on iron deficiency and development has an overall increasing trend, dominated by the United States, China, and India. The top five publishing journals included Nutrients, Journal of Nutrition, and American Journal of Clinical Nutrition, with the Journal of Nutrition having the highest link strength. The frequently co-occurring keywords included iron, anemia, and iron-deficiency.

Although COVID-19 had some disruption on publication due to priority on the pandemic-related research, the overall global trend. The occurrence of nutrition journals suggests an emphasis on the nutritional aspects of managing iron deficiency. The recurring keywords highlight the data focus primarily on early pediatric care, plant science, and medical research.

## Introduction and background

Iron is a necessary micronutrient for numerous key biological functions, including cellular growth, energy production, oxygen transport and utilization through hemoglobin, and neurological function [1]. Beyond poor blood health and red blood cell formation, inadequate iron intake during early development may impair cognitive development, decrease work productivity and efficiency, and could potentially impact the ongoing cycle of poverty in developing regions [2-4]. Globally, iron deficiency (ID) is ranked as one of the most common micronutrient deficiencies, particularly prevalent in low- and middle-income countries where malnutrition is widespread [2,3]. Over the past 30 years, the prevalence of iron deficiency has increased significantly, rising from 985 million cases in 1990 to over 1.27 billion in 2021; this figure is projected to further escalate to 1.44 billion by 2050 [2]. This trend underscores the importance of further research to establish interventions supporting iron-deficient populations.

Iron deficiency anemia (IDA), a more severe form of chronic iron deficiency, is the most palpable consequence of prolonged iron deficiency as a result of inadequate iron intake, poor absorption (malabsorption), and increased blood loss. Those vulnerable to this nutritional disorder include toddlers, women who are menstruating or pregnant, and individuals with cancer, gastrointestinal disorders, or heart failure [1,5]. Particularly alarming are the long-term neurobehavioral effects associated with early-life iron deficiency, including permanent alterations in dopamine metabolism and structural and functional changes in the hippocampus and frontal lobe of the brain, even after iron repletion and treatment [3]. ID present in children just under two years old can impair their learning and academic performance in the long term [3].

Despite affecting over 30% of the world according to the World Health Organization (WHO), this micronutrient deficiency is frequently underdiagnosed or overlooked as the majority of symptoms are vague or nonspecific [2,6]. Early signs may include dizziness, fatigue, irritability, exercise intolerance, and pallor. Severe iron deficiency can present with tachycardia, shortness of breath, and diaphoresis [3]. Accordingly, numerous children, older adults, and pregnant women remain under-treated or receive treatment after the condition progresses to stages of further severity [7]. Accurately predicting the future burden of iron deficiency is crucial for guiding effective public health interventions and resource allocation.

Despite the growing interest in iron deficiency for global health discussions, there is a notable underrepresentation of studies focusing on its impact on development in pediatric populations, particularly in developed regions where malnutrition is a growing concern. [1]. This study utilizes a bibliometric approach to visualize and analyze these trends, specifically with the connection between iron deficiency and human developmental outcomes. By identifying these trends and gaps, this analysis can redirect future efforts to nutrition advocacy for children in underdeveloped countries.

## Review

Methods

Data Source and Search Strategy

For this study, a dataset was obtained from the Web of Science (WoS) Core Collection database, which encompasses scientific articles from the twentieth century to the present day from multidisciplinary journals, conferences, and other scientific documents. This search was limited to the topic field, comprising the abstract, keywords, and title. Due to its extensive composition of high-quality publications, the WoS core database is considered to be the most optimal for bibliometric analysis [8,9].

Literature was collected from the years 1965 to 2025. In the WoS core database, the search terms “iron deficiency” and “development” were utilized to create the following query: “iron deficiency (Topic) and development (Topic).” No other limitations, such as punctuation and capitalization, were added to the search term, and there was no exclusion based on language or type of article, as all document types related to the search term were analyzed. The retrieved publications were exported in groups of 500 documents at a time as tab-delimited text files, which included the full record and cited references of each article. After filtering out and excluding articles that did not meet the inclusion criteria for the period, the following data were extracted and analyzed: title, abstract, author, keywords, publishing journal, country of publication, and the number of citations.

Data Analysis

Bibliometric analysis is a valuable quantitative method utilized to graphically represent and evaluate scientific literature. Researchers can assess publication patterns and research trends to guide their understanding within a specific area of research and stimulate further inquiry through identifying gaps [8,9]. This study uses a bibliometric approach to analyze the trends in publications regarding iron deficiency and human development. Descriptive statistics are reported. Inferential statistics were not applied, as the goal of this work was to report on the landscape of the existing literature.

From the WoS core collection, all tab-delimited text files were imported into VOSViewer software (version 1.6.2) for analysis and trend collection. VOSViewer is a data visualization tool that creates visual bibliometric maps to explore data relationships related to five types of analysis: bibliographic coupling, cartographic analysis, co-occurrence of keywords, co-citation, and co-authorship. VOSViewer represents each item as a circle, its size depicting its significance or contribution. Items are connected by corresponding colored links, in which the lengths and thicknesses determine the strength of the connection between them (also known as total link strength) [8,10].

Results

Publication Trends

A total of 6,850 publications were found on the Web of Science platform relating to iron deficiency and development within the study period of 1965-2025. Of these publications, 5,123 were original research articles (74.8%), 1,498 were review articles (21.9%), followed by 317 conference proceedings papers (4.6%); the remaining forms of publications included editorials, book chapters, and other formats.

**Figure 1:**
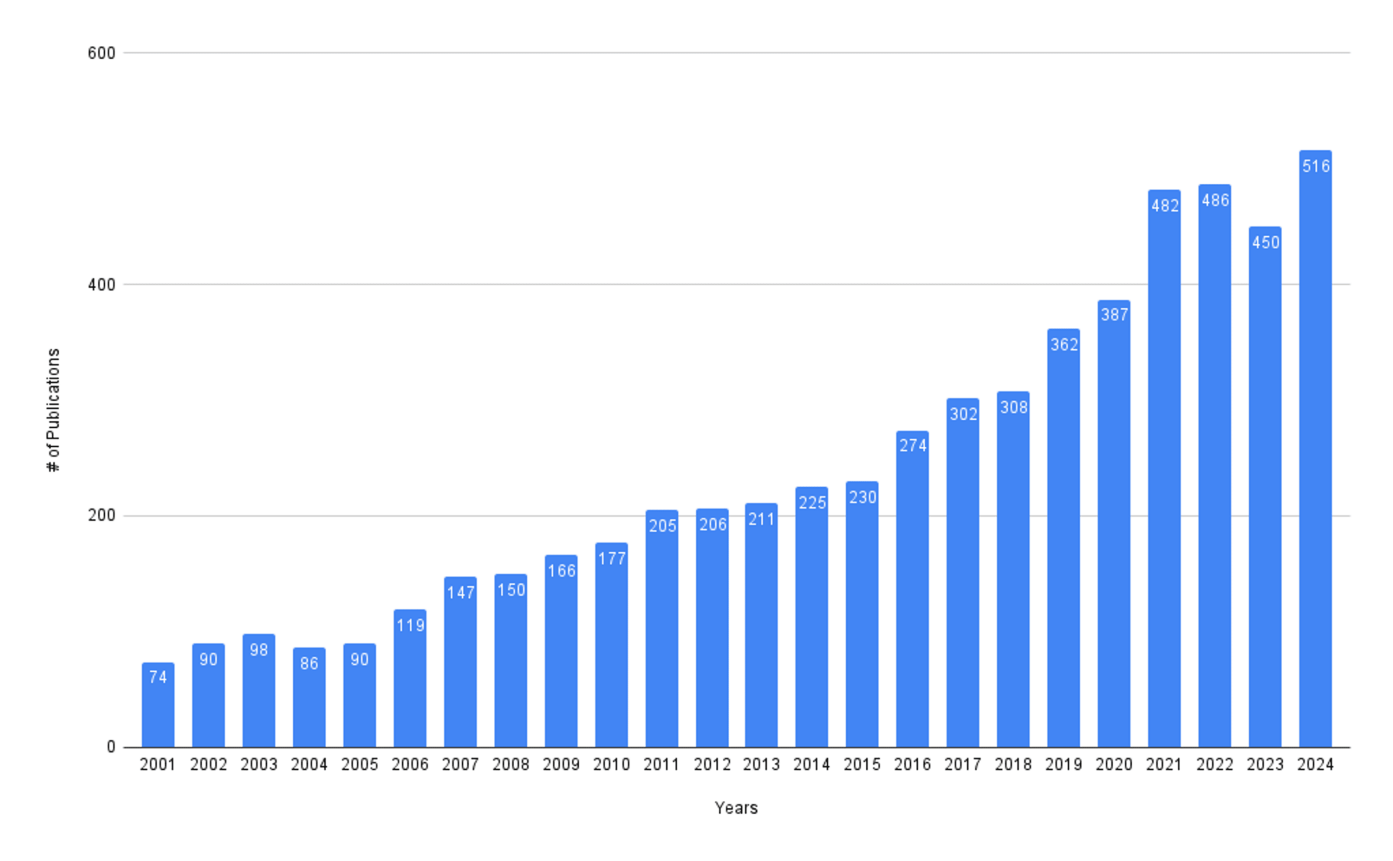
Annual growth of publications on iron deficiency and development from 2001 to 2024

As depicted in Figure 1, the number of publications over the past two decades has progressively increased, indicating a growing interest in iron deficiency and development. Despite the slight decline from 2021 to 2023, the overall observed trend represents a continued interest in iron deficiency and development.

Geographic Distribution

**Figure 2:**
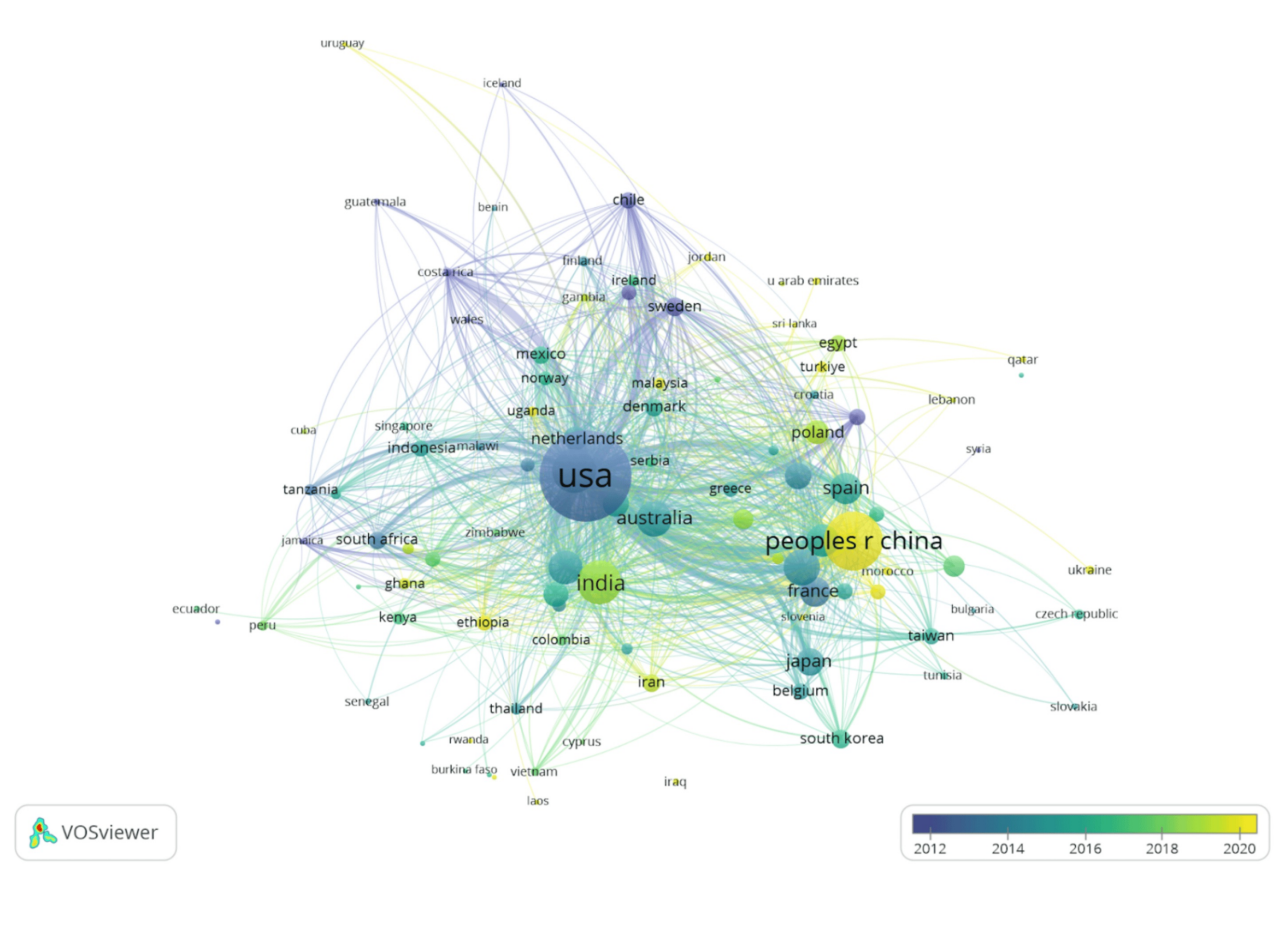
Global publication output and collaboration by region

Figure 2 displays the distribution of countries contributing to publications regarding iron deficiency and development. The most productive countries in terms of the highest number of publications are the USA (2044), the People’s Republic of China (864), India (487), and the United Kingdom (468). These countries contribute 3,395 articles, which account for 49.6% of all publications in the current literature on this topic. The United States had the largest number of international collaborations, with an average of 17,831 link strengths. However, it is worth noting that the People’s Republic of China (link strength of 4909) has been most active as of recent times within the global research network, shown with a yellow color on the map, with an average publication year of 2019.9. More established countries projecting long-term contributions show bluer hues.

Journal Analysis

**Figure 3:**
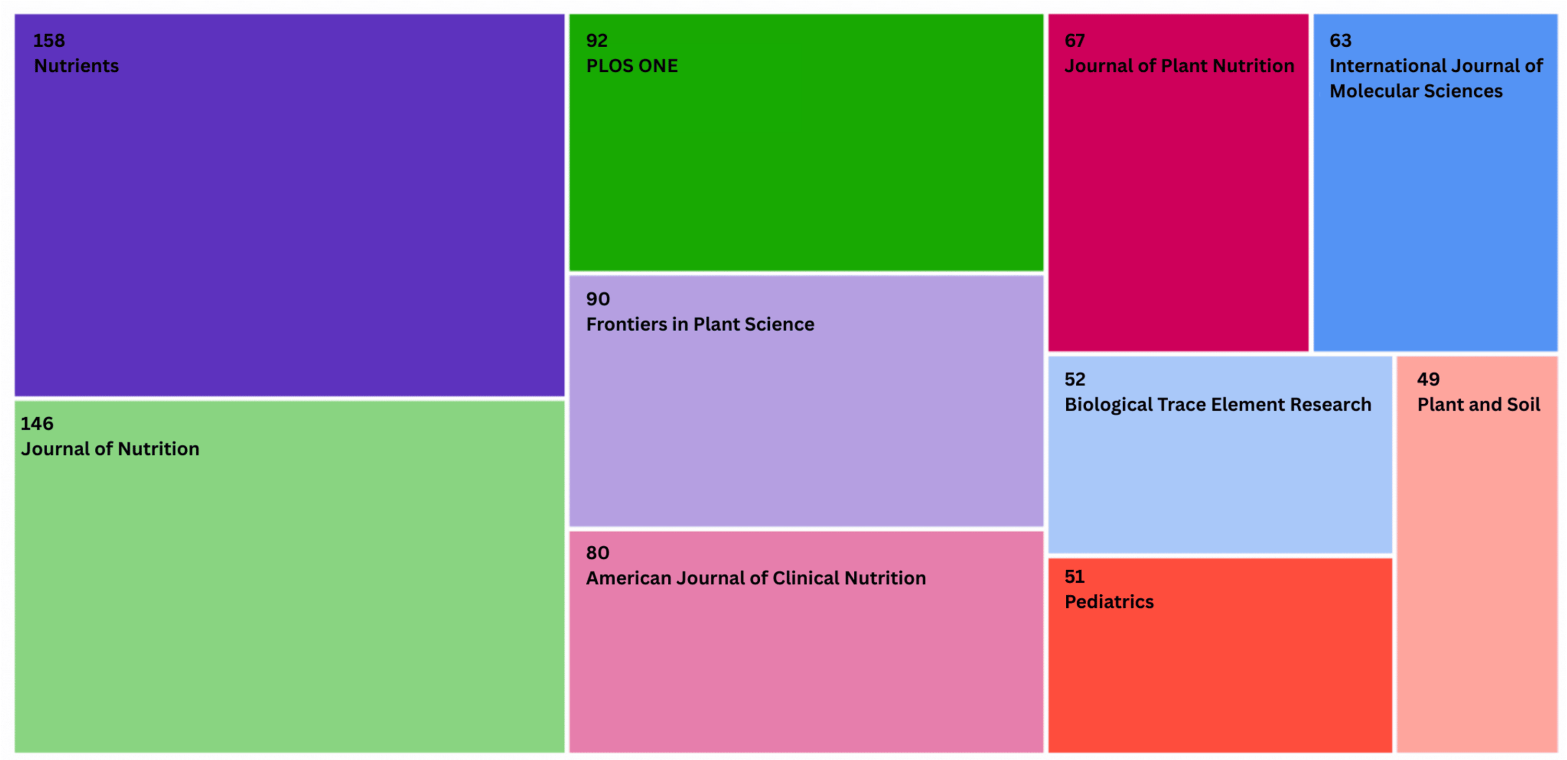
Top 10 journal publications by the number of articles

**Figure 4:**
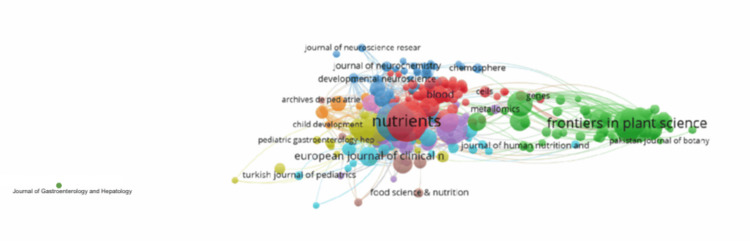
Most influential publishing journals

Figure 3 presents a treemap visualization of the top 10 journals publishing research on iron deficiency and development. Out of the 6,850 articles analyzed, a total of 848 were published in these journals, accounting for 12.3% of all publications related to the topic. The journal Nutrients contributed the highest number of publications (158), followed by the Journal of Nutrition (146), Plos One (92), Frontiers in Plant Science (90), and American Journal of Clinical Nutrition (80) as the fifth highest rank. However, based on Figure 4, the Journal of Nutrition has the greatest citations and total link strength of 1831 and 10481, respectively.

Keyword Analysis

**Figure 5:**
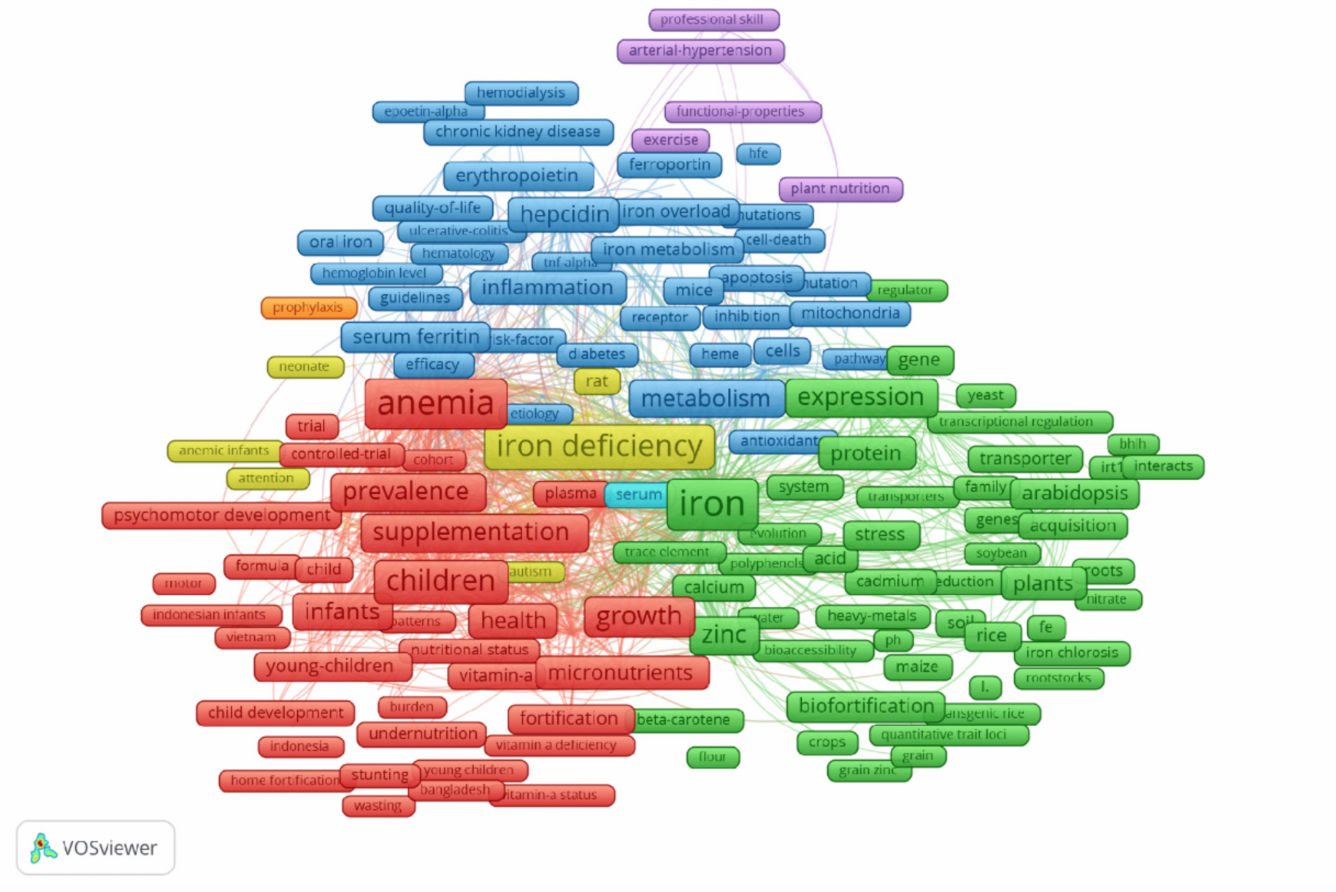
Keyword frequency in iron deficiency and development research

Figure 5 displays the themes of the most common keywords found in the literature. The keyword “iron” is the most frequently occurring keyword, appearing in 24.8% of the top 5 keyword occurrences, followed by “anemia” (21.8%), “iron-deficiency” (20.4%), “deficiency” (18.7%), and “iron deficiency” (14.4%).

Discussion

As established, iron deficiency is a micronutrient disorder that can impair cognitive, emotional, and physical human development, especially during early life stages [3,11]. This makes it an essential concern to be addressed in current healthcare protocols according to the World Health Organization [3,12]. In this article, a bibliometric analysis was conducted to visually analyze patterns using a computer mapping software in 6,850 publications on this topic, compiled from WoS from 1965 to 2025. Analysis of the current literature includes the publication output, countries, journals, organizations, and frequently used keywords.

The surge in publications over the past two decades demonstrates iron deficiency as a growing health concern. The slight decrease in publications from 2021 to 2023 can be attributed to the shift toward research focus on COVID-19-related publications. As journals began to prioritize publications on the pandemic, early studies from 2019-2020 have shown an estimated 18% reduction in non-COVID-19 publications during the pandemic, along with the reduction of non-COVID MeSH terms overall [13,14]. Though these studies are prior to the period of decline in Figure 1, it is probable to infer that this disruption extended throughout the pandemic, contributing to the period of decline in iron deficiency research until 2024, when the upward trend reappeared.

The analyzed literature shows a majority in the form of research articles, review articles, and conference proceedings papers. These articles also dominated the top 100 cited papers on iron deficiency in 2021, which suggests their importance and relevance in research on this topic [15]. Original research articles provide detailed primary data on iron deficiency in populations and are rated high for newsworthiness, review articles provide possible interventions based on the given data and are rated high for relevance, and proceedings papers provide discussion on the preliminary results of research through collaboration [16,17]. These forms of publications can advance data collection and analysis on iron deficiency in development, opening up discussions for public health interventions in iron-deficient populations.

A total of 6,850 publications were published in 156 different countries and regions, and this number will likely increase over time based on the trend in Figure 1. This bibliometric analysis highlighted that the countries with the highest contributions were the United States, the People’s Republic of China, and India, accounting for nearly half of all publications (Figure 2). The United States has the highest total link strength, which reflects its leading role in international collaboration and output. China has also experienced a major surge in publications as of 2019, likely due to increased funding. India has one of the highest burdens of anemia on a national level, which can explain its high representation and priority of iron deficiency research [18]. Notably, a majority of developing countries have the resources and funding that can be designated to such research regarding supplemental nutrient deficiency, whereas other countries must allocate their funding to other, more pressing issues or problems [19]. This disparity suggests the need for increased research investment in low- to middle-income countries where there is a higher risk of iron deficiency, as this can ensure the most affected communities can receive equitable health outcomes.

Over 5.6% of publications from Nutrients, Journal of Nutrition, and the American Journal of Clinical Nutrition (three of the top five journals publishing this topic) account for the total literature (Figure 3). Iron deficiency is commonly associated with blood health, but dietary factors can also play a major role in development and management. This emphasizes the role of journals with a focus on nutrition in iron deficiency and development, as overall dietary patterns and an insufficient intake of adequate iron (mainly in vulnerable countries where the bioavailability of iron is limited) are instrumental in the development of the micronutrient disorder [20]. It is also proven that certain dietary measures can help manage iron deficiency, such as increased Vitamin C and iron consumption to enhance absorption for anaemic women, and managing dietary practices [21-23]. For the treatment and prevention of iron deficiency, research in leading nutrition journals highlights the importance of nutrition in development.

Additionally, with the influence and prominence of these top journals, the developmental consequences of iron deficiency can be discussed to inform and support global healthcare interventions for integrating nutritional interventions for iron deficiency management. The Journal of Nutrition, as shown in Figure 4, has a high citation and link strength, indicating that it is frequently referenced by other articles on the same topic. The Journal of Nutrition has been proven to maintain a high impact factor and citation rates with a major influence in the nutrition science field [24,25]. This further suggests the key role that nutrition research has in developmental health outcomes surrounding iron deficiency.

The growing importance of understanding iron deficiency and its effects on development can be illustrated by the frequency and high link strength of keywords related to the topic, such as “iron” and “anemia,” as described in Figure 5. These are important terms utilized in the field of current research related to the topic. The bibliometric analysis of the articles identified four primary data clusters or focus areas based on the current literature. The largest cluster in red includes keywords such as anemia, prevalence, children, infants, supplementation, and growth; these terms focus mainly on public health and pediatrics in the context of iron deficiency. The keywords in this cluster explain the importance of iron deficiency interventions during early development, which has been established to impair neurocognitive, psychomotor, and physical development in newborn infants born to iron-deficient mothers, as well as children and adolescents [25,26]. The green cluster concentrates mainly on plant science and nutrition through the inclusion of words such as iron, expression, zinc, biofortification, and plants. Iron and zinc biofortification of crops has been an approach to alleviating iron deficiency through genetic engineering, which aids in developing countries where there is a low bioavailability of iron [27]. With terms involving hepcidin, metabolism, and inflammation, the third primary cluster in blue involves research on iron deficiency on a molecular and biological/medical level, such as the role of hepcidin and the inflammatory impacts on iron absorption in the body, which has been of growing interest [27]. The pattern displayed in Figure 5 is consistent with currently published bibliometric studies, which show that the most cited and interconnected topics in iron deficiency research involve clinical aspects, nutritional interventions, and molecular research. The connection between these clustered keywords can provide a depth of understanding in iron deficiency research and development.

With regards to understanding iron deficiency research and development, a study examining the 2021 data from the Global Burden of Diseases, Injuries, and Risk Factors Study (GBD) provides timely context [28]. The authors note that dietary iron deficiency remains a major health concern with a global prevalence of 16.7%. This is particularly true for women, children, and residents in low socio-demographic index countries.

## Conclusions

This bibliometric analysis summarizes the state of the published literature on iron deficiency and development. Although there is a disproportionate impact on women, children, and those from low sociodemographic index countries, there is a lack of scholarly contributions from developing countries, where malnutrition and iron deficiency are most prevalent. This suggests the need for greater global collaboration to inform interventions in high-risk populations.

## References

[1] Lynch S, Pfeiffer CM, Georgieff MK (2018). Biomarkers of Nutrition for Development (BOND)--iron review. J Nutr.

[2] Wang L, Liang D, Huangfu H (2024). Iron deficiency: global trends and projections from 1990 to 2050. Nutrients.

[3] Leung AK, Lam JM, Wong AH, Hon KL, Li X (2024). Iron deficiency anemia: an updated review. Curr Pediatr Rev.

[4] Radlowski EC, Johnson RW (2013). Perinatal iron deficiency and neurocognitive development. Front Hum Neurosci.

[5] Georgieff MK (2011). Long-term brain and behavioral consequences of early iron deficiency. Nutr Rev.

[6] Kumar A, Sharma E, Marley A, Samaan MA, Brookes MJ (2022). Iron deficiency anaemia: pathophysiology, assessment, practical management. BMJ Open Gastroenterol.

[7] Benson AE, Lo JO, Achebe MO (2025). Management of iron deficiency in children, adults, and pregnant individuals: evidence-based and expert consensus recommendations. Lancet Haematol.

[8] Manoj Kumar L, George RJ, Anisha PS (2023). Bibliometric analysis for medical research. Indian J Psychol Med.

[9] Ganti L, Persaud NA, Stead TS (2025). Bibliometric analysis methods for the medical literature. Acad Med Surg.

[10] Ullah F, Shen L, Shah SH (2022). Value co-creation in business-to-business context: a bibliometric analysis using HistCite and VOS viewer. Front Psychol.

[11] Pivina L, Semenova Y, Doşa MD, Dauletyarova M, Bjørklund G (2019). Iron deficiency, cognitive functions, and neurobehavioral disorders in children. J Mol Neurosci.

[12] Pasricha SR, Tye-Din J, Muckenthaler MU, Swinkels DW (2021). Iron deficiency. Lancet.

[13] Raynaud M, Goutaudier V, Louis K (2021). Impact of the COVID-19 pandemic on publication dynamics and non-COVID-19 research production. BMC Med Res Methodol.

[14] Riccaboni M, Verginer L (2022). The impact of the COVID-19 pandemic on scientific research in the life sciences. PLoS One.

[15] Frater JL (2021). The top 100 cited papers in the field of iron deficiency in humans: a bibliometric study. Biomed Res Int.

[16] Wanner A (2018). Why publish scientific meeting proceedings?. Chronic Obstr Pulm Dis.

[17] McKinlay RJ, Cotoi C, Wilczynski NL, Haynes RB (2008). Systematic reviews and original articles differ in relevance, novelty, and use in an evidence-based service for physicians: PLUS project. J Clin Epidemiol.

[18] Petersen OH (2021). Inequality of research funding between different countries and regions is a serious problem for global science. Function (Oxf).

[19] Skolmowska D, Głąbska D, Kołota A, Guzek D (2022). Effectiveness of dietary interventions to treat iron-deficiency anemia in women: a systematic review of randomized controlled trials. Nutrients.

[20] Chouraqui JP (2022). Dietary approaches to iron deficiency prevention in childhood-a critical public health issue. Nutrients.

[21] Jani N, Keshteli AH, Kabiri P (2012). A 10-year performance trajectory of top nutrition journals and impact factors. J Res Med Sci.

[22] Lei XG (2025). Highlights of The Journal of Nutrition in 2024 ahead to 2025. J Nutr.

[23] Means RT (2020). Iron deficiency and iron deficiency anemia: implications and impact in pregnancy, fetal development, and early childhood parameters. Nutrients.

[24] Mattiello V, Schmugge M, Hengartner H, von der Weid N, Renella R (2020). Diagnosis and management of iron deficiency in children with or without anemia: consensus recommendations of the SPOG Pediatric Hematology Working Group. Eur J Pediatr.

[25] Connorton JM, Balk J (2019). Iron biofortification of staple crops: lessons and challenges in plant genetics. Plant Cell Physiol.

[26] Beck KL, Heath AL (2013). Dietary approaches to assessing iron-related nutrition. Curr Opin Clin Nutr Metab Care.

[27] Ueda N, Takasawa K (2018). Impact of inflammation on ferritin, hepcidin and the management of iron deficiency anemia in chronic kidney disease. Nutrients.

[28] Lee S, Son Y, Hwang J, Kim MS, Il Shin J, Yon DK, Kassebaum NJ (2025). Global, regional and national burden of dietary iron deficiency from 1990 to 2021: a Global Burden of Disease study. Nat Med.

